# Application of exosomes as liquid biopsy in clinical diagnosis

**DOI:** 10.1038/s41392-020-00258-9

**Published:** 2020-08-03

**Authors:** Biting Zhou, Kailun Xu, Xi Zheng, Ting Chen, Jian Wang, Yongmao Song, Yingkuan Shao, Shu Zheng

**Affiliations:** 1 Cancer Institute, Key Laboratory of Cancer Prevention and Intervention, Ministry of Education, The Second Affiliated Hospital, Zhejiang University School of Medicine, 310009 Hangzhou, Zhejiang China; 2 Department of Colorectal Surgery and Oncology, Key Laboratory of Cancer Prevention and Intervention, Ministry of Education, The Second Affiliated Hospital, Zhejiang University School of Medicine, 310009 Hangzhou, Zhejiang China

**Keywords:** Molecular biology, Tumour biomarkers

## Abstract

Liquid biopsy refers to the sampling and molecular analysis of the biofluids of circulating tumor cells, extracellular vesicles, nucleic acids, and so forth. Exosomes are small extracellular vesicles with sizes between 30–150 nm. They are secreted by multivesicular bodies through exocytosis in live cells and can participate in intercellular communication due to their contents, including nucleic acids, proteins, and lipids. Herein, we investigate publication frequencies on exosomes over the past 10 years, and review recent clinical studies on liquid biopsy of exosomes in the fields of oncology, pregnancy disorders, cardiovascular diseases, and organ transplantation. We also describe the advantages of exosomes as an effective liquid biopsy tool and the progression of exosome extraction methods. Finally, we depict the commercial development of exosome research and discuss the future role of exosomes in liquid biopsy.

## Introduction

Exosomes, which are small extracellular vesicles with sizes between 30–150 nm, are a subtype of extracellular vesicles (EVs), which are secreted by all cell types and are responsible for intercell communication.^1^ The International Society for Extracellular Vesicles (ISEV) recommends using “EVs” instead of exosomes or microvesicles and gives terms for EV subtypes based on the physical properties.^2^ We quoted the original statement out of respect for their research. However, most articles cited in this review did not clarify the definition clearly and were only capable of enriching rather than purifying exosomes with current methods. Exosomes were originally described to be released from sheep reticulocytes.^3^ With the large number of studies that followed, exosomes were found to exist in almost all body fluids, primarily blood,^4^ urine,^5^ cerebrospinal fluid,^6^ saliva,^7^ pleural effusion,^8^ ascites fluid,^9^ amniotic fluid,^10^ breast milk,^11^ and bronchoalveolar lavage fluid (BALF).^12^ Exosomes, originating from the endosomal pathway via the formation of late endosomes or multivesicular bodies,^1^ enclose a variable spectrum of molecules characterized by parent cells, including nucleic acids (DNA, mRNA, microRNA (miRNA), lncRNA, circRNA, etc.), proteins, and lipids, which can be transported over distances within the protection of a lipid bilayer-enclosed structure.^1^ Recently, a contradictory point was raised in a study published in *Cell* that based on proteomic profiles, double-stranded DNA and DNA-binding histones were absent in exosomes or any other type of small EVs.^13^ An autophagy- and multivesicular endosome-related pathway was the driver of extracellular DNA secretion instead of exosome-dependent pathway.^13^ However, Yokoi et al.^14^ subsequently confirmed the presence of double-stranded DNA in exosomes by imaging flow cytometry and described how nuclear content loaded into exosomes. Since the DNA detection methods applied in these studies are different, it is unclear whether genomic DNA exists in exosomes, although we still recognize the value of the studies on exosomal DNA (exoDNA).

**Fig. 1:**
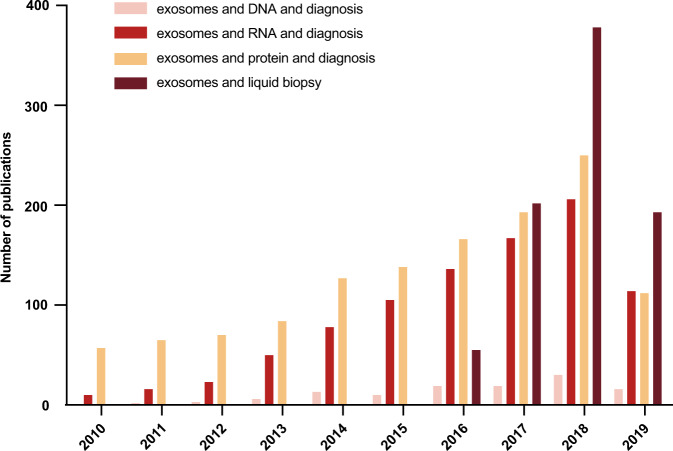
Publication frequencies of studies investigating different contents of exosomes as liquid biopsy for disease diagnosis over the past 10 years based on the PubMed search, January 2020

Researchers around the world express great enthusiasm for exosomes as biomarkers in liquid biopsy. Based on a PubMed search in January 2020, we statistically analyzed the number of publications regarding the diagnostic efficacy of exosomes in the past 5 years with the MeSh Terms “exosomes” OR “small extracellular vesicles” (microparticles and microvesicles are not MeSh Terms in PubMed), “diagnosis” OR “biomarker”, “mutation” OR “copy number” OR “DNA”, “RNA” (“mRNA”, “microRNA”, “lncRNA”, “circRNA”), “protein”, and “liquid biopsy”. There were 88 relevant publications on DNA, 695 on RNA (including 74 about mRNA, 534 about microRNA, 52 about lncRNA, 14 about circRNA), and 824 on protein. Protein is the most-studied content of exosomes, followed by miRNA. It attracted the greatest interest in exosome research as a biomarker carrier for diagnosis over the past 10 years, especially in 2018 (Fig. 1).

**Fig. 2:**
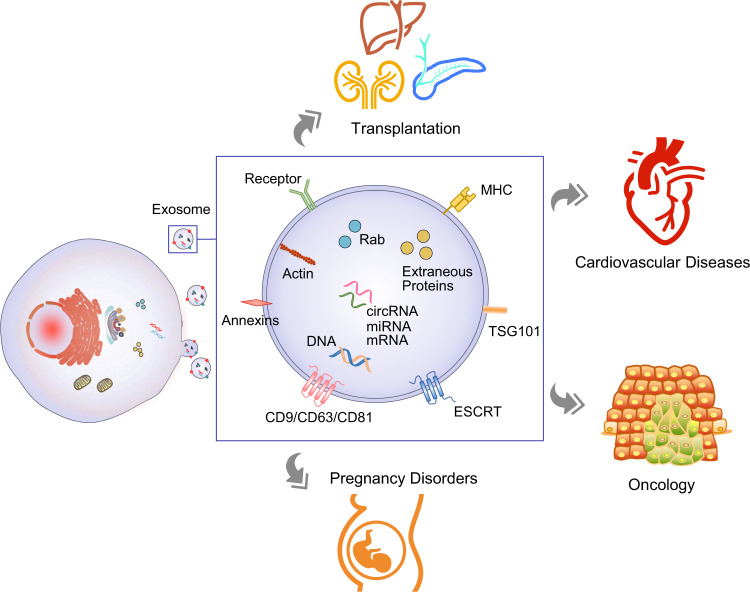
Biogenesis, secretion, composition, and application of exosomes as liquid biopsy. Exosomes, originating from the endosomal pathway via the formation of late endosomes or multivesicular bodies, enclose a variable spectrum of molecules characterized by parent cells, including nucleic acids (DNA, mRNA, miRNA, lncRNA, circRNA, etc.), proteins, and lipids, which shows great promise in clinical applications in cancer, pregnancy disorders, cardiovascular diseases and organ transplantation

Simultaneously, exosomes play critical roles in various physiological and pathological processes, including cancer, pregnancy disorders, cardiovascular diseases, and immune responses.^15^ By virtue of the exponential evolution of liquid biopsy in recent decades, traditional solid biopsy shows considerably more limitations. It is imperative to introduce liquid biopsy to clinical practice to reduce invasive operations and promote more precise medical intervention.^16^ Herein, we mainly introduce the advantages of exosomes as liquid biopsy and their application as a potential complement to personalized medicine in some common malignant tumors, pregnancy disorders, cardiovascular diseases, and organ transplantation (Fig. 2). Owing to the great prospects of exosomes in clinical applications, a commercial chain of exosome research-related technologies has been formed and is still under development.

## Advantages of exosomes in liquid biopsy

Exosomes show significant superiority over other sources of liquid biopsy. First, exosomes exist in almost all body fluids and possess high stability encapsulated by lipid bilayers. Similar quality of exosomal markers exists in samples stored at 4 °C for 24 h and then at −80 °C, samples immediately stored at −80 °C and fresh urine samples.^17^ And it is better to keep exosomes at 4 °C within 24 h but at −80 °C for long-term storage. Exosomes prefer to be isolated in pH 7 solution rather than in acidic environments.^18^ The high biological stability can reduce the cost of sample short-term storage and the difficulty of transportation, which greatly enhances the clinical applicability of exosomes. Second, exosomes are secreted by living cells and contain biological information from the parental cells and be more representative than cell-free DNA (cfDNA), which is secreted during necrosis or apoptosis.^19^ Third, exosome identification is clear and simple. Exosomes express specific proteins such as CD63, ALIX, TS101, and HSP70,^20^ which can be used as markers to effectively distinguish exosomes from other vesicles, and their specific cup-shaped characteristic makes them identifiable by electron microscopy.^21^ Fourth, exosomes can present specific surface proteins from parental cells^22^ and even target cells, which can realize the isolation of origin-specific exosomes and predict organ-specific metastasis.^23^ Fifth, exosomes show superiority to conventional serum-based biomarkers, such as carcinoembryonic antigen, in diagnostic accuracy.^24^ Sixth, compared with circulating tumor cells (CTCs), exosomes are relatively convenient to obtain from almost any body fluids. Additionally, there are many recognized classic extraction methods, such as ultracentrifugation,^25^ and a considerable number of novel methods under development, for instance, commercial isolation kits, indicating more feasibility for clinical application than CTCs, while CTC collection is still tough and complicated.^26^ In addition, with regard to cfDNA, most human plasma cfDNA is located in exosomes,^27^ and the copy number of mitochondrial DNA is higher in exosomes than in plasma or peripheral blood from patients with advanced serous epithelial ovarian cancer.^28^ Meanwhile, the detection sensitivity and specificity of exoDNA mutation frequency are higher than those of cfDNA^29–31^ and exoDNA has shown greater prognostic value as well.^29,32^ Generally, despite the slightly more complicated DNA extraction procedures, exosomal DNA possesses more abundant biological information and higher accuracy for prognosis prediction than cfDNA.

## Exosomes in tumor diagnosis, prognosis prediction, and treatment response assessment

At present, solid biopsy is still the gold standard for pathological diagnosis and is mostly the basis for treatment of cancer. However, solid biopsy is invasive, sometimes unable to perform, and tumor heterogeneity is inevitable. Noninvasive liquid biopsy shows great advantages for individualized and precise diagnosis and treatment.^16^ Tumor-derived exosomes (TDEs) are critically related to tumor progression, metastatic niche formation, and immune evasion,^33^ which indicates that TDEs may hold great promise for cancer diagnosis, prognosis and treatment response assessment.

### Exosomes in early tumor diagnosis

Early screening and accurate diagnosis are undoubtedly the primary issues for patients with tumors or precancerous lesions in reducing mortality and increasing the recovery rate. In pancreatic cancer, a high probability of *KRAS* mutation in circulating exoDNA was found in the early-stage.^29^ Given that the elevated level of GPC1^+^-circulating exosomes obviously occurred in patients with pancreatic ductal carcinoma (PDAC)^34^ and colorectal cancer (CRC)^35^ compared to healthy controls, this could serve as an early detection tool for tumors in the digestive system. A clinical trial (NCT03032913) conducted by Etienne BUSCAIL completed the recruitment of 20 PDAC patients and 20 noncancer patients, whose blood samples were collected to detect CTCs and GPC1^+^ exosomes for diagnosis accuracy assessment and comparison. Diverse forms of exosomal RNAs also promisingly take part in the early diagnosis of cancers. A panel consisting of two mRNAs (KRTAP5-4 and MAGEA3) and one lncRNA (BCAR4) was a promising candidate for the CRC diagnosis.^36^ A database of exosome-containing RNA (including 18,333 mRNAs, 15,501 lncRNAs, and 58,330 circRNAs) in human blood provided a platform for further discovery and clinical application of circulating exosomal biomarkers.^37^ In lung cancer, detection of exosome-based *EGFR T790M* has shown great potential for clinical diagnosis to avoid unnecessary tumor biopsies in non-small cell lung cancer (NSCLC).^38^ Multiple proteins in exosomes exert powerful efficacy in distinguishing cancerous and noncancerous patients. By using an EV array containing 49 antibodies that could capture and detect exosomes in plasma, Sandfeld-Paulsen et al.^39^ revealed that CD151, CD171, and tetraspanin 8 were the most significant molecules to separate patients with all histological lung cancer from cancer-free individuals. Of note, based on the development of mass spectrometry (MS) technology and proteome profiles, thousands of proteins can be captured from one sample of micro quantity. Chen et al.^40^ used MS to compare breast cancer (BC) patient-derived exosomes with those of noncancer patients and identified 144 distinctly elevated exosome phosphorylated proteins. Subsequently, they utilized MS and parallel reaction monitoring techniques to validate four of them: PKG1, RALGAPA2, NFX1, and TJP2. In further study and clinical application of liquid biopsy, the combination of various contents of exosomes as biomarkers can provide a more effective guarantee for the accuracy of cancer diagnosis and prognosis.^41,42^ The studies described above primarily focused on peripheral blood-derived exosomes, while as common malignant tumors, urinary system- and genitourinary tract-related cancers are preferentially detected through urine-based testing.^43^ In renal cell carcinoma, after MS profiling and a literature search, Raimondo et al.^44^ selected ten proteins as a panel to distinguish cancer and healthy control. Furthermore, multiple miRNAs carried by exosomes, such as miR-21-5p,^45,46^ miR-4454 and miR-720/3007a, were elevated in bladder cancer patient urine,^46^ which could potentially serve as early diagnosis biomarkers for bladder cancer.

### Exosomes in tumor prognosis prediction

With longitudinal monitoring in the treatment course of patients with metastatic PDAC by plasma-based exoDNA detection, Bernard et al.^32^ indicated that *KRAS* mutation detected at baseline by digital droplet PCR (ddPCR) and a mutation frequency above 5% indicated poor clinical outcome. In another study, exosomal *KRAS* mutations were proven to be better than CA 19-9 levels for the prognostic surveillance of patients with PDAC.^29^ Keklikoglou et al.^47^ revealed why cytotoxic chemotherapy promotes metastasis in BC. They found that annexin A6-enriched exosomes were largely secreted after cytotoxic chemotherapy and transferred to endothelial cells (ECs) in the lung, thus inducing a premetastatic niche to lay the foundation of lung metastasis. Exosome-associated Annexin II^48^ and L-plastin^49^ also play a vital role in metastasis and can be potential candidates for the prognosis of advanced BC. In lung cancer, FLI1 exonic circular RNAs was identified as a novel carcinogenic driver contributing to the metastasis of small cell lung cancer (SCLC), which can be a potential biomarker for the prognosis and surveillance of SCLC.^50^ In prostate cancer, based on the MS proteomic profile, Bijnsdorp et al.^51^ revealed that urine-derived exosomal ITGA3 and ITGB1 were upregulated in metastatic patients compared with those with benign tumors and early-stage cancer. Recently, it was revealed that circulating exosomal PD-L1, but not soluble PD-L1, was associated with tumor progression in head and neck cancer^52^ and NSCLC.^53^ Simultaneously, serum-based exosomal PD-L1 possessed the ability to predict unfavorable prognosis in PDAC.^54^

**Fig. 3:**
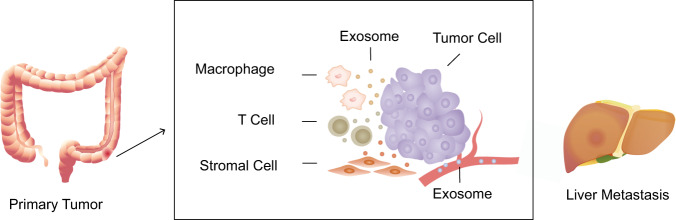
Exosomes in liver metastasis of colorectal cancer. Stromal cell-derived exosomes can carry bioactive molecules (e.g., miR-92a-3p) to CRC cells and enhance epithelial-mesenchymal transition and cell stemness to promote liver metastasis. Many other miRNAs (e.g., miR-193a, miR-21, miR-25-3p, miR-18a, miR-17-5p, miR-141-3p, miR-548c-5p, miR-375, and miR-6803-5p) encapsulated in exosomes from primary CRC cells, which flow to the liver via blood circulation and lead to liver metastasis, can be candidates for prognosis

Liver metastasis of colorectal cancer is still a major problem that needs to be overcome. Multiple works have been conducted to determine the mechanism of liver metastasis, including the exosome-mediated hypothesis. Teng et al.^55^ demonstrated that miR-193a, a tumor suppressor miRNA, was selectively sorted into TDEs by major vault protein, which led to higher expression of oncogenic miRNAs in the tumor cells and promoted colon cancer progression. Overexpression of miR-193a in circulating exosomes can be a promising biomarker for the liver metastasis in colon cancer. Zeng et al.^56^ illustrated that TDE miR-25-3p regulated the liver metastasis of CRC by inducing vascular permeability and angiogenesis and thus creating a premetastatic niche. A prospective clinical trial on rectal cancer was carried out and found that plasma-based exosomal miR-141-3p and miR-375 were significantly increased in patients with liver metastasis compared to those without.^57^ Recently, Hu et al.^58^ revealed another mechanism of liver metastasis of CRC via cancer-associated fibroblast-derived exosomes, which elevated the expression of miR-92a-3p and enhanced epithelial-mesenchymal transition and cell stemness in CRC cells. Complementarily, miR-21,^59^ miR-18a, miR-17-5p,^60^ and miR‐548c‐5p^61^ can be used as early screening markers for liver metastasis of CRC (Fig. 3). In summary, miRNAs are critical elements in regulating liver metastasis of colorectal cancer, whereas the specific targets are still under exploration, and an accurate exosomal miRNA panel for prognosis prediction makes sense in clinical applications.

### Exosomes in tumor treatment response assessment

Exosomes also play a potentially useful role in treatment response assessment, especially drug resistance, which is a major obstacle for advanced malignant tumors. TDEs can carry various drug resistance-associated molecules to target cells^62^ and thus induce EMT, promote antiapoptotic pathways, alter signal transduction alterations, and regulate specific targets in cancer cells^63^ to facilitate treatment failure. Wei et al.^64^ demonstrated that exosomal miR-222-3p could promote the expression of gemcitabine resistance and a malignant phenotype by targeting the promoter of SOCS3, which could be used as a predictor of treatment response for NSCLC patients. Qu et al.^65^ found a novel lncRNA-lncARSR (lncRNA activated in renal cell carcinoma with sunitinib resistance), as a biomarker for treatment, as it is transferred through tumor-derived exosomes to promote sunitinib resistance by upregulating AXL and c-MET expression. Nevertheless, there are also some favorable contents that enhance sensitivity to chemotherapy. Liu et al.^66^ revealed that miR-128-3p-containing exosomes derived from normal intestinal cells would be transported to oxaliplatin-resistant CRC cells and thus increase the treatment response. Upregulated exosomal miR-567 could reverse trastuzumab resistance in BC as well.^67^ Another mechanism of the drug resistance is the drug‐efflux ability of cancer cells via exosomes to decrease intracellular drug accumulation, including direct drug export^68^ and transfer of drug efflux pumps (e.g., P-glycoproteins, ATP-transporter A2/A3, multidrug-resistant protein-1).^69^ Importantly, tumor-associated macrophage-derived exosomes also contribute to drug resistance by targeting cancer cells to inactivate sensitivity to therapy.^70,71^

On the other hand, as a hotspot in cancer therapy, exosomal PD-L1 has attracted considerable attention from researchers for its potential in anti-PD-1/PD-L1 therapy response prediction. In melanoma, upregulated by interferon-γ, an increasing number of PD-L1-containing exosomes were secreted by malignant cells, indicating resistance to anti-PD-1 therapy in patients without previous immunotherapy but denoting a favorable response in patients on pembrolizumab treatment since nonresponsive patients hardly experienced changes in exosomal PD-L1 levels during immunotherapy.^72^ To date, most studies on cancer therapy response assessment remain at the level of cancer cells in vitro, and more clinical trials are needed to validate the clinical capacity of these biomarkers (Table 1).

**Table 1:** Exosomes in common malignant tumors

Applications	No. of patients	Source	Volume of body fluid	Targets	Exosome extraction	Extraction method	Detection method	References
Early diagnosis								
Pancreatic cancer	263	Plasma	0.9–1.5 ml	*KRAS*	Ultracentrifugation	MagAttract High Molecular Weight DNA kit	ddPCR	^29^
	221	Serum	250 μl	GPC1	Ultracentrifugation + sucrose gradient centrifugation	Affinity capture	Flow cytometry analysis	^34^
	85	Serum	–	CKAP4	PS Capture Exosome ELISA Kit	PS Capture Exosome ELISA Kit	PS Capture Exosome ELISA Kit	^170^
Colorectal cancer	140	Serum	300 μl	lncRNA	Ultracentrifugation	TRIzol	qPCR	^36^
	40	Serum	250 μl	miRNA	ExoQuick-TC™ Exosome Precipitation Solution kit	miRNeasy Serum/Plasma kit	qRT–PCR	^160^
	102	Tissue homogenate	–	GPC1	ExoCap^TM^ Exosome Isolation, Enrichment kit	Affinity capture	Flow cytometry analysis	^35^
		Plasma		miR‐96‐5p, miR‐149		TRIzol	qPCR	
	116	Serum	250 μl	CEA	ExoQuick^TM^ Exosome Precipitation Solution	ELISA	ELISA	^171^
	124	Plasma	500 μl	Copine III	Ultracentrifugation	PIPA buffer	ELISA	^24^
Lung cancer	210	Plasma	1–2 ml	*EGFR T790M*	Ultracentrifugation	ExoLution Plus platform	Allele-specific qPCR assay	^38^
	105	Plasma	–	miRNAs	Ultracentrifugation	mirVanaTM miRNA Isolation Kit	qPCR	^172^
		BALF						
	581	Plasma	–	Proteins	Extracellular vesicle array	Extracellular vesicle array	Extracellular vesicle array	^39^
	171	Serum	–	Proteins	Ultracentrifugation	Lysis buffer	Immunoblotting	^173^
Breast cancer	32	Plasma	250 μl	miR-21, miR-1246	Exoquick-TC^TM^ reagent	TRIzol	qRT–PCR	^174^
	38	Serum	–	miR-105	Ultracentrifugation	TRIzol	qRT–PCR	^175^
	240	Serum or Plasma	500 μl	CD82	ExoQuick^TM^ exosome precipitation solution	Strong RIPA lysate	ELISA/western blot	^176^
	44	Plasma	5.5 ml	Phosphoproteins	Ultracentrifugation	–	LC-MS/MS	^40^
Renal cell carcinoma	52	Urine	–	Proteins	Differential centrifugation + density gradient ultracentrifugation/ultrafiltration	–	LC-MS/MS	^44^
Bladder cancer	69	Urine	38.5 ml	miRNAs	Differential centrifugation	miRNeasy Mini Kit/RNA MS2/Clean-up Kit	miRNA microarray	^45^
Cholangio-carcinoma	134	Serum	1 ml	Proteins	Ultracentrifugation	–	MS	^177^
Prognosis								
Pancreatic cancer	194	Plasma	15 ml	*KRAS*	Ultracentrifugation	QIAmp Circulating Nucleic Acid Kit	ddPCR	^32^
	91	Serum	250 μl	PD-L1, c-MET	Invitrogen Total Exosome Isolation Reagent	–	Flow cytometry	^54^
Colorectal cancer	255	Serum	1 ml	miR-19a	Ultracentrifugation/Total Exosome Isolation Kit	miRNeasy mini kit	qRT–PCR	^178^
	28	Serum	1 ml	miR-17-5p, miR-92a-3p	qEV Size Exclusion Columns	HiPure Liquid RNA, miRNA Kit	qRT–PCR	^60^
	108	Serum	100 μl–1 ml	miR‐548c‐5p	Invitrogen™ Total Exosome Isolation Kit	miRNeasy mini kit	qPCR	^61^
	93	Plasma	500 μl /1 ml	miR-141-3p miR-375	Exiqon^TM^ miRCURY Exosome Isolation Kit	Exiqon^TM^ miRCURY RNA Isolation Kit	qPCR	^57^
	87	Serum	–	miR-25-3p	Ultracentrifugation	TRIzol	qPCR	^56^
Lung cancer	106	Serum	up to 4 ml	FLI1 exonic circular RNAs	ExoEasy Maxi Kit	TRIzol	qRT–PCR	^50^
	276	Plasma	10 μl	NY‐ESO‐1	Extracellular Vesicle Array	Extracellular Vesicle Array	Extracellular Vesicle Array	^179^
	85	Serum	4 ml	PD-L1	Invitrogen™ Total Exosome Isolation Kit	RIPA	ELISA	^53^
	20	Plasma	5 ml	Amphiregulin	Ultracentrifugation	–	ELISA	^180^
Head and neck cancer	40	Plasma	1 ml	PD-L1	Mini size exclusion chromatography	–	Flow cytometry	^52^
Breast cancer	53	Serum	5 ml	miR-222	Density gradient centrifugation	Maxwell® 16 miRNA Tissue kit	qPCR	^181^
Melanoma	56	Serum	200 μl	miRNA-125b	ExoQuick^TM^ precipitation solution	TRIzol	qPCR	^155^
	96	Serum	250 μl	S100B, MIA	ExoQuick^TM^ precipitation solution	–	Immuno assays	^182^
Esophageal carcinoma	602	Saliva	3–5 ml	Transcriptionally induced chimeric RNAs	ExoQuick™ exosomes precipitation solution	TRIzol	qRT–PCR	^183^
Prostate cancer	13	Urine	5 ml	ITGA3, ITGB1	Ultracentrifugation	Lysis buffer	Western blot	^51^
Treatment response								
Lung cancer	84	Plasma	3 ml	EGFR (RNA)	ExoLution™ Plus	ExoLution™ Plus	NGS	^41^
	50	Serum	–	miR-222-3p	Ultracentrifugation	RNeasy Kit	PCR	^64^
Breast cancer	53	Serum	5 ml	miR-21	Density gradient centrifugation	Maxwell® 16 miRNA Tissue kit	qPCR	^181^
Melanoma	–	Plasma	1 ml	PD-L1	Ultracentrifugation + exosome isolation kit	Reverse phase protein array	Reverse phase protein array	^72^

Recent clinical studies on exosomes as biomarkers for early diagnosis, prognosis and treatment response monitoring of cancers, including cohort scale, source of body fluid, functional targets of exosomes, exosome extraction methods, target isolation and detection methods

## Exosomes in pregnancy disorders

Exosomes show great capacity for the diagnosis of pregnancy disorders, including hypertension and hyperglycemia during gravidity, and prenatal screening. Although the utilization of exosomes in maternal peripheral blood, urine and amniotic fluid during pregnancy is still under exploration, the established facts that placental cells can release exosomes to communicate with the maternal body and the biogenesis and secretion of placenta-derived exosomes (PDEs) are regulated by the microenvironment, such as glucose concentration and oxygen tension,^73^ make exosomes a noninvasive and promising tool for the early diagnosis and prognosis of pregnancy disorders.

### Exosomes in hypertensive disorders of pregnancy

Hypertensive disorders of pregnancy, especially preeclampsia (PE) and eclampsia, are the major risks for the health of women and their infants.^74^ PE affects 3–5% of pregnancies, leading to severe maternal-fetal mortality.^75^ Placental hypoxia, which can be caused by PE, increases the secretion of exosomes from placental cells and varies the components.^76^ In addition to conventional markers, placental alkaline phosphatase (PLAP) is a placenta-specific marker for the extraction and quantification of PDEs.^77,78^ Pillay et al.^79^ demonstrated that the ratio of PDEs to the total number of exosomes (PLAP^+^ exosome ratio) was strikingly reduced in early-onset PE and late-onset PE, whereas the relative concentration of PDEs was significantly increased compared to that in normotensive patients. Biro et al.^80^ collected plasma samples from pregnant women diagnosed with PE, gestational hypertension or chronic hypertension, and healthy controls. Plasma-based exosomal miRNA analysis through reverse transcription polymerase chain reaction (RT-PCR) revealed that the levels of total miRNA and hypoxia-sensitive miR-210 in circulating exosomes were markedly higher in the PE patients than other groups, especially in severe PE. A study on placental exosome changes in PE women across gestation conducted by Carlos Salomon identified exosomal miRNAs by next generation sequencing and finally found that miR-486-1-5p, miR-486-2-5p, and exosome concentration were strikingly higher in PE than in healthy controls and that the two miRNAs selected could serve as potential candidates to predict the occurrence of PE,^81^ which could greatly improve the management of pregnancy hypertension.

### Exosomes in gestational diabetes mellitus

Hyperglycemia is another major factor affecting the bioactivity of placental exosomes. Insulin resistance and hyperinsulinemia can be induced by pregnancy-related hormones (e.g., diabetogenic autacoids) in normal pregnancies.^82,83^ Gestational diabetes mellitus (GDM) is characterized as glucose intolerance for the first diagnosis during pregnancy.^84^ GDM affects up to 25% of pregnancies worldwide,^85^ and the incidence is rapidly increasing due to the global increase in type 2 diabetes and obesity.^86^ The release and bioactivity of PDEs are regulated by glucose in the first trimester of gestation.^87^ Salomon et al.^88^ carried out a retrospectively stratified study on the changes in PDEs in pregnant women with GDM during gestation. They established that the level of PDEs was elevated in both normal and GDM pregnancies, while higher in GDM, especially in early gestation (11–14 weeks). Early diagnosis of GDM and timely pharmacological interventions could reduce the long-term damage on mothers and fetuses.^89^

### Exosomes in prenatal screening

As well, the detection of exosomes can play a role in prenatal screening. PLAP^+^ exosome ratio from maternal plasma was a potential marker of fetal growth and placental function, as it was obviously lower in patients with fetal growth restriction than in healthy controls.^90^ Combined with type-B ultrasonic examination and physical examination, plasma exosome detection could allow much more precise diagnosis and monitoring of fetal growth restriction before parturition. Additionally, downregulated miR-300 and miR-299-5p in amniotic fluid-based exosomes could serve as biomarkers for the diagnosis of congenital obstructive nephropathy. Regrettably, invasive amniocentesis remained.^91^

In summary, maternal circulating exosomal miRNAs, particularly PDEs, are the most popular biomarkers studied in pregnancy disorders, prenatal screening and preterm birth monitoring.^92^ Urinary exosomes were also affected by maternal changes in gestation, which possessed potential for the diagnosis of intrahepatic cholestasis^93^ and hypertension.^94^ Blood and urine testing are already regular during pregnancy, and exosomal biomarkers could be a supplement to detect and predict disorders in pregnant women and fetuses.

## Exosomes in cardiovascular diseases

Cardiovascular diseases (CVDs) are one of the major concerns in human health, especially coronary artery disease (CAD), which remains the leading cause of global mortality.^95^ Currently, circulating biomarkers of CVDs, such as total cholesterol levels and low-density lipoproteins (LDL), and myocardial infarction (MI) prognostic biomarkers, including high-sensitivity C-reactive protein, high-sensitivity cardiac troponin and creatine kinase MB, can only roughly evaluate the risk of the occurrence and progression of the disease but cannot precisely predict whether the process starts or develops.^96,97^ In this sense, novel blood-based exosome biopsy can offer a promising platform to assist clinical diagnosis and prediction more accurately. Mounting works have proven that exosomal miRNAs possess promising protective functions in CVDs.^98–100^ Simultaneously, circulating exosomes have shown great potential for diagnosis and risk assessment in CVDs.^95^

### Exosomes in coronary artery disease

In CAD, exosomal miR-133a was elevated in injured myocardium and dead cardiomyocytes in particular.^101^ Furthermore, miR-146a-abounded exosomes from cardiosphere-derived cells were revealed to promote angiogenesis and inhibit apoptosis, which indicates the therapeutic efficacy of exosomes.^102^ Exosomes containing miR-210, miR-132, miR-181,^103^ and miR-378b, miR-623, miR-941 (associated with ejection fraction improvement), miR-1256, miR-384 (associated with fibrosis reduction), miR-525-3p, miR-5155p, miR-1224 (associated with angiogenesis induction)^104^ and GATA4-responsive-miR-451^105^ derived from cardiac progenitor cells also possess the same cardioprotective function. Recently, Liu et al.^106^ made substantial progress in identifying the therapeutic role of circulating endothelial cell-derived microvesicle miRNAs, particularly miR-92a-3p, in regulating the phenotypes of ECs and vascular smooth muscle cells under atherosclerotic conditions, which could be a candidate for the prognosis of CAD. In contrast, miR-939-5p was downregulated in serum-based exosomes from patients with MI and inhibited angiogenesis via the nitric oxide signaling pathway.^107^ Apart from miRNAs, exosomal proteins also play a significant role in CVD. P-selectin-expressing microparticles,^108^ CD3^+^/CD45^+^, SMA-α^+^-circulating exosome levels,^109^ and exosomal Cystatin C, Serpin F2, CD14 levels^110^ were correlated with a high risk for incident CVD and mortality. Based on liquid chromatography coupled to tandem mass spectrometry (LC-MS/MS), Cheow et al.^111^ identified 252 upregulated EV proteins after MI and created a potential panel for the early diagnosis of MI, including apolipoprotein C-III, apolipoprotein D, platelet glycoprotein Ib alpha chain, complement C1q subcomponent subunit A, and complement C5.

### Exosomes in heart failure

In heart failure (HF), miR-22, miR-320a, miR-423-5p, and miR-92b were overexpressed in both serum and serum exosomes and can be uesd as specific biomarkers for the diagnosis and prognosis of systolic HF.^112^ Several serum-based exosomes containing p53-responsive miRNAs, such as miR-34a, miR-192, and miR-194, were upregulated in HF patients within 1 year of acute MI onset.^113^ Moreover, it was demonstrated that an increased ratio of endothelial apoptotic microparticles (CD31^+^/Annexin V^+^) to mononuclear progenitor cells was related to adverse clinical outcome in patients with acutely decompensated chronic HF.^114^

In addition, exosomes are also related to multiple other CVDs, such as stroke,^115^ cardiomyopathy,^116,117^ cardiac arrhythmia,^118^ and valvular heart disease.^119^ Generally, miRNAs are the most prevalent molecules in CVD-associated exosomes as well, revealing superiority as a diagnostic biomarker and a promising therapeutic tool.

## Exosomes in organ transplantation

Transplantation of organs from living donors is a feasible way to cure patients with advanced organ failure. However, the recognition of the allograft by the recipients’ immune system, and thus the subsequent rejection, is a major obstacle in organ transplantation therapy. Allograft rejection is mediated by T lymphocytes in the recipient’s secondary lymphoid organs that recognize donor major histocompatibility (MHC) antigens through direct (donor MHC and peptides) or indirect (recipient MHC and donor-derived peptides) pathways.^120^ The activation of T lymphocytes is ascribed to the migration of the donor antigen from professional antigen-presenting cells to recipient lymphoid tissues and directly communication with T lymphocytes.^121^ In recent years, it has been discovered that allograft rejection depends on the transmission of EVs from the donor graft organ, which carries donor MHC to recipient antigen-presenting cells and activates the immune response to allografts.^122^ The T-cell activation pathway via exosomes is termed the semidirect pathway.^123,124^ Accurate and early diagnosis and longitudinal monitoring of immunologic rejection are essential in the prevention and treatment of clinical transplantation. Current detection methods can sense immune rejection, but by then, the transplanted organ has often undergone irreversible damage. Consequently, there is an urgent need for convenient and noninvasive tools to detect chronic allograft rejection, which affects the survival of most transplant recipients. Thus, the existence of donor-specific exosomes and exosomal changes caused by immunologic rejection over time^125^ can serve as a surrogate biomarker in acute or chronic rejection of solid organ allografts.

### Exosomes in lung transplantation

In lung transplantation, serum/BALF-based exosomes presenting donor HLA, SAgs and immunoregulatory miRNAs from recipients might contribute to acute rejection (AR) and predict early diagnosis of allograft rejection.^126^ Besides, analysis of BALF-based exosomal mRNAs was significantly different between lung transplantation recipients with or without AR, and the upregulated molecules in AR samples showed a substantial trend toward an inflammatory environment related to both innate and adaptive immune responses.^127^

### Exosomes in heart transplantation

In heart transplantation, Kennel et al.^128^ demonstrated that circulating exosomal protein content varied in heart transplantation recipients with allograft rejection and that fifteen proteins were strikingly different and primarily related to the immune response. Consequently, exosomal protein analysis could be a powerful tool for post-transplantation monitoring. Besides, Sukma et al.^129^ demonstrated a new view on the mechanism of acute cellular rejection in cardiac allografts in which miR-142-3p-incorporated exosomes from heart transplant patients were transferred to ECs and undermined endothelial barrier function via downregulation of RAB11FIP2. Recently, Saha et al.^104^ proved the protective role of cardiac progenitor cell-derived exosomes in MI. They demonstrated that quantitative and cargo profiles of exosomes from circulating cardiac progenitor cells or cardiosphere-derived cells could be a potential tool for noninvasive surveillance after heart transplantation.

### Exosomes in kidney transplantation

In kidney transplantation (KTx), Park et al.^130^ developed a detective and analytic method–integrated kidney exosome analysis (iKEA). iKEA showed high detection accuracy in clinical urine samples from patients with kidney transplant rejection, and its portability simplifies transplant recipient monitoring. Recently, Lim et al.^131^ conducted a proteomic analysis of urinary exosomes derived from KTx patients. Based on MS, 169 urinary exosome proteins were identified, among which 46 were upregulated in stable recipients and 17 were overexpressed in AR patients. Finally, they selected tetraspanin-1 and hemopexin, which were remarkably elevated in patients with AR, as potential markers for the diagnosis of AR in KTx recipients. Plasma exosomes also play a role in KTx. Zhang et al.^132^ demonstrated that plasma exosome mRNA-based analysis can be a potential tool for the early diagnosis of allograft rejection in KTx patients.

### Exosomes in islet transplantation

In islet transplantation, Vallabhajosyula et al.^125^ demonstrated that proteomic and RNA signatures, quantity and other signal changes in donor HLA exosomes could indicate early injury/loss of islet mass; therefore, transplant islet exosomes could be a reliable biomarker for monitoring patients undergoing islet transplantation over long-term follow-up. Recently, Korutla et al.^133^ reported a relevant case confirming that circulating transplant islet-specific exosomes could be a potential biomarker for distinguishing between pancreatic β cell damage secondary to autoimmune relapse or immune rejection in islet-transplanted patients diagnosed with autoimmune type 1 diabetes.

**Table 2:** Exosomes in pregnancy disorders, cardiovascular diseases, and organ transplantation

Fields	No. of patients	Source	Volume of body fluid	Targets	Exosome extraction	Extraction method	Detection method	Findings	References
Pregnancy disorders									
Pregnancy hypertension	98	Plasma	500 μl	miR-210	Exosome precipitation solution	NucleoSpin miRNA Plasma Kit	qPCR	Diagnosis	^80^
Preeclampsia	47	Plasma	1 ml	miR-486-1-5p, miR-486-2-5p	Ultracentrifugation + ultrafiltration + OptiPrep gradient centrifugation	miRNeasy Mini Kit, TRIzol	NGS	Diagnosis	^81^
	45	Plasma	1 ml	PLAP	Ultracentrifugation	ELISA	ELISA	Diagnosis	^79^
Preterm birth	20	Plasma	1 ml	miRNAs	Ultracentrifugation	RNeasy Mini Kit	NGS	Prognosis	^92^
Gestational diabetes mellitus	20	Plasma	1 ml	CD63, PLAP	Ultracentrifugation + ultrafiltration + OptiPrep gradient centrifugation	ELISA	ELISA	Prognosis	^88^
Congenital obstructive nephropathy	8	Amniotic fluid	10 ml	miR-300, miR-299-5p	Ultracentrifugation + sucrose gradient centrifugation	TRIzol	miRNA microarray	Diagnosis	^91^
Intrauterine growth restriction	30	Plasma	1 ml	PLAP	Ultracentrifugation + iodixanol gradient centrifugation	Quantum dots	ELISA	Diagnosis	^90^
Cardiovascular diseases									
Myocardial infarction	20	Serum	1 ml	miR-939	Ultracentrifugation	TRIzol	qPCR	Prognosis	^107^
	35	Plasma	5 ml	proteins	Ultracentrifugation	Trypsin	LC-MS/MS	Diagnosis	^111^
Coronary artery disease	180	Plasma	250 μl	miR-92a-3p	Ultracentrifugation	TRIzol	qPCR	Prognosis	^106^
Vascular disease	1060	Plasma	150 μl	Cystatin C, Serpin F2, CD14	ExoQuick™	Roche Complete Lysis-M	Immunoassay	Prognosis	^110^
Heart failure	20	Serum	–	miR-423-5p, miR-22, miR-320a, miR-92b	ExoQuick™ Exosome Precipitation Solution	Ethanol precipitation	qRT–PCR	Prognosis	^112^
	100	Serum	250 μl	miRNA	ExoQuick™ Exosome Precipitation Solution	ISOGEN II	qPCR	Prognosis	^113^
Stroke	131	Serum	–	miR-9, miR-124	ExoQuick^TM^ Solution	Exosome RNA Purification Kit	qPCR	Diagnosis	^115^
Cardiac arrhythmia	37	Serum	30 μl	IL-1 β, P-selectin	Affinity capture	ELISA	ELISA	Prognosis	^118^
Organ transplantation									
Lung	30	BALF	–	SAgs, Collagen-V	Ultracentrifugation	–	Western blot	Diagnosis	^126^
		Serum	1 ml		Total Exosome Isolation Reagent kit				
	12	BALF	20-60 ml	mRNAs	Ultracentrifugation	miRNeasy, miRCURY	RNA-Seq	Diagnosis	^127^
Heart	10	Serum	–	miR-142-3p	Ultracentrifugation	microRNeasy mini kit	qRT–PCR	Diagnosis	^129^
	48	Serum	200 μl	Proteins	Total Exosome Isolation	Trypsin	LC-MS/MS	Prognosis	^128^
Kidney	44	Urine	15 ml	CD3	Ultracentrifugation	iKEA	iKEA	Prognosis	^130^
	47	Urine	–	Tetraspanin-1, Hemopexin	Ultracentrifugation	RIPA buffer	LC-MS/MS	Diagnosis	^131^
	64	Plasma	–	mRNAs	exoRNeasy Serum/Plasma Midi Kit	exoRNeasy Serum/Plasma Midi Kit	qPCR	Diagnosis	^132^

Cohort scale, source of body fluid, functional targets of exosomes, exosome extraction methods, target isolation, and detection methods of recent clinical studies on exosomes as biomarkers for diagnosis or prognosis of these diseases

In conclusion, immune response-related proteins and immunoregulatory miRNAs varied significantly in exosomes derived from patients after organ transplantation. Peripheral blood and urine-based exosomes may serve as a practical tool for the early detection of AR and long-term monitoring to prevent chronic allograft rejection (Table 2).

## Progression in exosome extraction methods

Thery et al.^25^ firstly proposed the classic method of ultracentrifugation for isolating exosomes. After that, differential ultracentrifugation became the most common method for exosome isolation. According to a global survey in October 2015, >80% of researchers used this method for EV separation.^134^ Exosome extraction methods are mainly based on their physical characteristics and components.^16^ Importantly, exosome quality will be greatly affected by different exosome extraction procedure.^135^ Currently, these methods for exosome extraction can be commonly divided into differential ultracentrifugation, density gradient/cushion centrifugation, size exclusion chromatography (SEC), precipitation, (immuno-) affinity capture, microfluidic approaches, etc.^136^ While differential ultracentrifugation is the most commonly applied method for exosome isolation with relatively satisfactory purity, it is time-consuming and requires costly instruments, which are nevertheless disadvantages that cannot be ignored, making it untoward for clinical application.^137,138^ Density gradient centrifugation can further increase the exosome purity and has been recommended as a standard to validate an EV experiment,^139^ whereas it is more time consuming and of low yield.^2^ SEC excludes nonexosomal particles by vesicle size.^140^ However, some samples may contain a large proportion of lipoprotein particles of similar size to exosomes, and thus, they cannot be distinguished.^141^ Kaloyan et al.^142^ compared the difference between murine plasma exosomes extracted by ultracentrifugation and SEC. They found that the latter extracted a large number of exosomes that were enriched with protein but also with many chylomicron-positive lipid particles and nonvesicle-associated proteins.^142^ With a high yield of exosomes, the precipitation method provides the least pure exosomes compared to size exclusion isolation and density gradient purification.^143^ It often precipitates viruses, proteins, and other substances together with exosomes, which may influence subsequent experiments. In contrast, affinity capture methods often provide high purity, although the recovery seems unsatisfactory.^2^

As mentioned above, purity improvement is an issue that most methods need to consider, especially regarding lipoprotein contamination (including high-density lipoproteins (HDL), LDL, very low-density lipoproteins (VLDL), and chylomicrons) in blood samples.^140^ The similar density of EVs and HDL, as well as the tremendous gap in abundance between EVs and LDL, makes it difficult to separate them by density gradient ultracentrifugation. SEC can purify EVs from HDL/LDL by size differences, but not chylomicrons or VLDL, which possess similar sizes to EVs.^140,144^ The combination of ultracentrifugation and SEC^140^ or the combination of density gradient fractionation and immunoaffinity capture^13^ could greatly reduce contamination. Essentially, the most common way applied to decrease contamination is dilution, which is a necessary step before ultracentrifugation.^145^ What also caught our attention is that during the preparation of proteins/peptides for MS technology, a solid-phase extraction method will be utilized for lipoprotein removal,^146^ which may also be suitable for exosome purification. Minimal Information for Studies of Extracellular Vesicles guidelines (2018) put forward the classification of existing extraction methods based on recovery and specificity of exosomes,^2^ which provided a way for researchers to select appropriate and stable methods in subsequent studies. However, we are still going to explore a better methodology for substantial yields and reliable quality of exosomes.

Apart from these classic extraction methods, multiple new extraction and detection technologies have been reported in recent years, such as the microfluidic chip^147,148^ and a method of integrated extraction and quantitative analysis of exosomal nucleic acids and proteins,^149^ with high specificity and intact yield of exosomes, small required sample volume and simple, time-saving operation. Moreover, a novel urine-based EV extraction and enrichment method was established by Woo et al.^150^. This new technology takes only 30 min to enrich EVs from 4 ml urine. Following mRNA extraction, AR-V7 and androgen receptor full-length mRNA detection via ddPCR, they proposed exosomal AR-V7 transcript as a promising biomarker for the clinical application of urinary biopsy in prostate cancer.^150^ Additionally, a rapid and simple Vn96-peptide-based EV isolation method was established, which could capture heat shock proteins abounded on the surface of EVs.^151^ Bijnsdorp et al.^152^ optimized this time- (~1.5 h) and cost-efficient method and indicated similar efficacy compared to ultracentrifugation when extracting urinary EVs. At the same time, a size-based exosome total isolation chip, which was easy to operate, with higher yield and similar purity compared to ultracentrifugation and required small sample volumes (10–100 μl), was designed to simplify EV extraction from clinical biofluids, such as plasma, urine, and lavage.^153^ Complementarily, Chen et al.^154^ proposed an anion-exchange-based method for separating exosomes directly from plasma or cell culture media by anion magnetic beads within 30 min.

More importantly, the exosome isolation kit, the most representative commercial product, is becoming more prevalent recently and includes the ExoQuick precipitation solution,^155,156^ Total Exosome Isolation Reagent kit^126^ and ME™ kit^157^ based on precipitation; Exo-Spin™^158^ based on precipitation and size exclusion; and exoEasy Maxi Kit,^55^ ExoCap^TM^ Exosome Isolation and Enrichment kit, and Exo-Flow^TM^^159^ based on immunocapture. It has been reported that kit-based exosome isolation methods are more convenient and effective^160^ and obtain similar exosome recovery and purity comparable to the ultracentrifugation method.^128,161^ However, Tian et al.^138^ recently demonstrated that a large proportion of contaminants existed in products extracted by commercial isolation kits and that the products exhibited much lower purity than those extracted with ultracentrifugation. The use of commercial kits is still controversial, and more technical refinement is urged for clinical application. Finally, we summarized some cost-convenient exosome extraction methods, which possessed equivalent or higher exosome yield and purity compared with ultracentrifugation, potentially feasible for clinical use in Table 3.

**Table 3:** Exosome extraction methods potentially feasible for clinical application. Some novel and cost-convenient exosome extraction methods, which possessed equivalent or higher exosome yield and purity compared with ultracentrifugation, were summarized in terms of time, source, minimum volume of biofluid, exosome quality identification, technical principles and clinical field applied

Extraction method	Time	Source	Sample volume	Quality identification	Principle	Clinical field
Vn96-peptide-based	1.5 h	Plasma, culture media/urine	1 ml	Proteomics	Vn96-peptide binding to heat shock proteins- based affinity capture	Breast cancer^151^ Prostate cancer^152^ Nephronophthisis-related ciliopathies^184^
Exosome total isolation chip	5 ml/h (culture media), 1 h (plasma)	Culture media, plasma, urine, BALF	10–100 μl	miRNA sequence, proteomics	Nanoporous membrane-based filtration	Lung cancer^153^
Anion-exchange -based	30 min	Culture media, plasma	500 μl	Proteomics	Anion-exchange-based magnetic beads	Prostate cancer^154^
Microfluidic chip	Overnight for device functionalization, <20 min for capture and release	Culture media, serum	<100 μl	Proteomics	CD63/CD9 and EpCAM based immunoaffinity capture	Ovarian cancer^148,185^

## Commercial development of exosomes

A multitude of companies have been established to exploit biotechnology development in exosomes. Founded in 2015, Codiak BioSciences is a pioneer biotechnology company developing exosome treatments for various diseases and is headquartered in Cambridge, Massachusetts. Codiak BioSciences has developed the engEx™ Platform to engineer exosomes to express and deliver therapeutic drug candidates. ExoSTING is a major and promising immune therapeutic candidate targeting cancer. Compared to free STING agonists, exoSTING is highly potent with minimal toxic potential. It is rarely affected by serum systemic cytokines and preserves the vitality of effector T cells and antigen-presenting cells in tumors to maintain sustained immune protection. Recently, exoSTING is being developed for solid tumor therapy that activates the “STING” receptor in immune cells. Relevant clinical trials will be carried out in the first half of 2020.^162^ Exosome Diagnostics is a revolutionary developer of molecular diagnostics based on biological fluids, which was acquired by Bio-Techne last year. They are aimed at developing novel and precise exosome technology mainly in liquid biopsy of multiple cancers, including lung^38,41^ and prostate cancer.^163,164^ The ExoDx Prostate® (IntelliScore) (EPI) test is the star production of exosome diagnostics. This is a urine-based and completely noninvasive test designed to assist physicians in assessing whether an individual patient over 50 years old tested with 2–10 ng/ml prostate-specific antigen, which presenting for a needle biopsy, is at greater risk for high-grade prostate cancer; therefore, the patient can avoid unnecessary biopsy and, instead, continue to follow up.^163^ Moreover, the commercial exosome isolation kit ExoLution Plus Isolation Kit owned by Exosome Diagnostics is widely used in exosome research. Recently, Avalon GloboCare and its subsidiary Genexosome Technologies clarified the establishment and development of the first salivary-based exosome miRNA biomarker-miR-185 as a dual target for the diagnosis and treatment of oral cancer.^165^ Additionally, Avalon GloboCare is working on the identification of human angiogenic exosomes.^166^ PureTech Health^167^ collaborated with Roche to impel the advancement of technology for oral administration of antisense oligonucleotides with PureTech’s milk exosome-based technology^168^ to transform conventional intravenous injection therapy for improved efficacy and reduced toxicity.

## Conclusions

With the progression of precise and individual medicine, conventional solid biopsy has gradually shown considerable limitations, whereas the occurrence of liquid biopsy greatly makes up for it and provides a promising platform for noninvasive diagnosis and prognosis. Undoubtedly, exosomes play an important role in various physiological and pathological processes, and compelling evidence has proven exosomes to be a potential tool for clinical application, including liquid biopsy and therapy, with the powerful advantages of existence in all body fluids, stable biological activity, higher sensitivity and specificity in diagnosis and prognosis, and organotropic characteristics.^23^ Meanwhile, various sources of biofluid are applied in different diseases according to human anatomy and pathophysiology. For example, nervous system diseases will prefer cerebrospinal fluid,^169^ prostate cancer, and urinary system diseases will benefit more from urine and most solid tumors, pregnancy disorders and CVDs are prone to blood testing.

However, there are still some barriers between basic research and real clinical practice. First, a standardization of the classification and extraction method of exosomes for different body liquids is urgently needed. More efficient methods with a low biofluid volume requirement and high purity and yield are the foundation of subsequent applications. Second, the identification of specific subtypes of EVs is urgently needed, as different vesicles may exert various biological effects. Current methods to extract exosomes (as shown in Tables 1 and 2) are too diverse to confirm the purity of the product. Therefore, it is necessary to standardize the protocols and identification methods when attempting to use exosomes widely in clinical testing. Additionally, more reliable biomarkers should be confirmed. Although many molecules carried by exosomes have been documented to serve as potential biomarkers, little of them are qualified for application. It may be a better direction to validate documented biomarkers on a larger scale to create new panels for multiple fields. Last but not the least, as potential therapeutic cargo, the biological safety, targeted efficacy, and adverse effects of exosomes must be confirmed before clinical use.
